# Calf circumference predicts frailty in older adults: the Chinese longitudinal healthy longevity survey

**DOI:** 10.1186/s12877-022-03644-w

**Published:** 2022-12-05

**Authors:** Ke Ying Xu, Jun Jie Wang, Jing Chen, Xinxiu Zhao, Ling Fang Yuan, Qin Zhang

**Affiliations:** 1 Department of Geriatrics, The First Affiliated Hospital, School of Medicine, Zhejiang University, Zhejiang, Hangzhou China; 2 Key Laboratory of Diagnosis and Treatment of Aging and Physic-chemical Injury Diseases of Zhejiang Province, The First Affiliated Hospital, School of Medicine, Zhejiang University, Zhejiang, Hangzhou China; 3 Department of Psychiatry, Hangzhou Seventh People’s Hospital, Hangzhou, Zhejiang, China

**Keywords:** Calf circumference, Frailty, Old adults

## Abstract

**Background:**

Although frailty is a common geriatric syndrome in old adults, a simple method to assess the degree of frailty in a person has not yet been established. In this study we have tried to establish the association between calf circumference (CC) and frailty among older Chinese people.

**Methods:**

We used the data obtained from the 2014 edition of the Chinese Longitudinal Healthy Longevity Survey; 1216 participants aged ≥60 years were included for the study. Body mass index, CC and waist circumference measurement data, and laboratory test results were collected. Frailty status was measured using the frailty index (FI). Participants were then classified into non-frail (FI < 0.25) and frail (FI ≥ 0.25) groups.

**Results:**

There were 874 participants (71.9%) in the non-frail group and 342 (28.1%) in the frail group. The CC was significantly different between the two groups (31.54 ± 4.16 versus 28.04 ± 4.53, *P* < 0.001). Logistic regression analysis revealed that CC (odds ratio = 0.947, 95% confidence interval: 0.904–0.993, *P* = 0.023) was an independent impact factor associated with frailty. The CC value of 28.5 cm was considered the best cut-off value in women with area under the curve (AUC) was 0.732 (*P* < 0.001) and 29.5 cm in men with AUC was 0.592 (*P* = 0.004);We created a simple prediction model for frailty that included age,sex and CC:

$$\textrm{Logit}(P)=-9.756+0.123\ast \textrm{Age}-0.512\ast \textrm{Sex}\left(\textrm{man}=1,\textrm{woman}=0\right)-0.053\ast \textrm{CC},$$

*P* = e^logit(P)^ /1 + e^logit(P)^, and AUC is 0.849 (*P* < 0.001).

**Conclusions:**

CC is a convenient and predictable marker of frailty in older adults.

**Supplementary Information:**

The online version contains supplementary material available at 10.1186/s12877-022-03644-w.

## Background

Frailty is a multidimensional geriatric syndrome that leads to serious health problems across the world. Chinese population is aging at an unprecedented speed. The data of prevalence for frailty among community-dwelling old adults in our country is as follows: approximately 6, 15, and 25% of people are aged 65–74, 75–84 and ≥ 85 years, respectively [1]. The old adults with frailty may suffer fatigue, falls, fractures, and sarcopenia that increase the risk for disabilities and mortality [2].

The most frequently used instruments to assess frailty in clinical research are the Fried frailty phenotype and frailty index (FI) [2]. The Fried phenotype includes weakness, exhaustion, low physical activity, slowness, and unintentional weight loss. However, the FI is comprehensive and systematic. It includes evaluation methods defined by Rockwood, which have 30 to 70 symptoms, which relate deficit accumulation to the individuals’ risk of death. It includes, among others, factors like living ability, cognitive function, and disease and disability status [3]. The literature supports the fact that frail individuals have many complicating factors, some closely associated with age and inflammatory or nourishment markers [4]. How to find a way to easily predict frailty in old adults is the aim of our study.

Calf circumference (CC) is a simple and convenient measurement method applied both in the community and in hospitals. The Asian Working Group for Sarcopenia 2019 recommends the use of CC as a method to diagnose sarcopenia because of its high sensitivity and specificity in the Asian population [5–7]. According to previous studies, a lot of diseases have close links to CC, such as sarcopenia, disability, non-alcoholic fatty liver, type 2 diabetes, and so on [8–10]. Moreover, CC can predict nutritional risks in old adults and forecast mortality among patients [11, 12]. So far, no study has reported the relation of CC and frailty.

In our research, we wanted to find a method to assess frailty using a simple tool. Therefore, we explored the association between CC and frailty among old people by gathering data from the Chinese Longitudinal Healthy Longevity Survey (CLHLS).

## Methods

### Study population

**Fig. 1:**
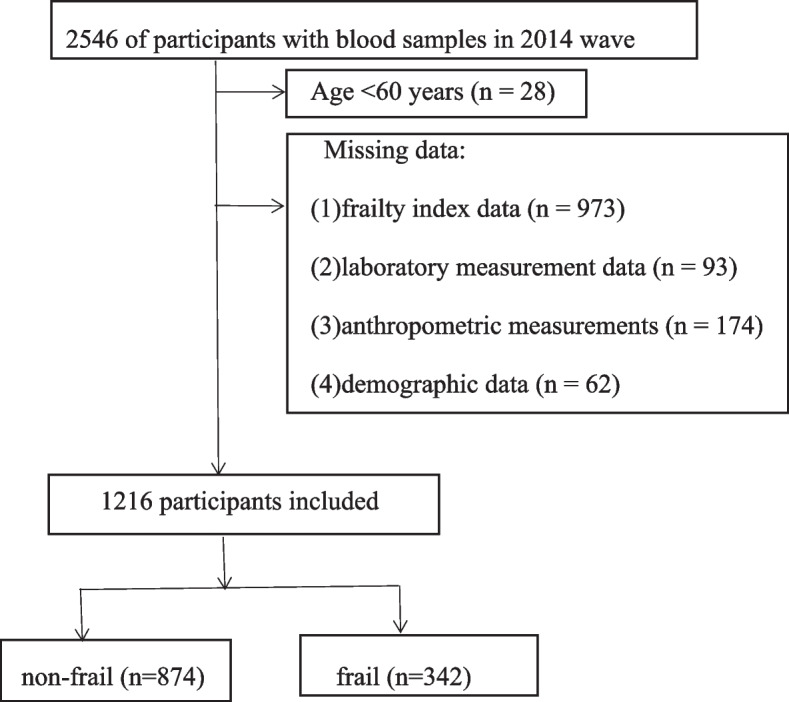
Flowchart of the study identification process

The CLHLS study is utilized to investigate health conditions through face-to-face interviews in the Chinese communities. The CLHLS study was established in 1998 and is conducted every 3 years in seven waves. It includes many cities in 23 out of 31 provinces and covers more than 85% of China’s population. For the present research, we have used the data obtained from the seventh wave of the CLHLS conducted in 2014. This survey included 7192 old adults; 2546 of them had blood samples collected for laboratory studies. In our study, the exclusion criteria were (1) Age < 60 years (*n* = 28); (2) Missing frailty index data (*n* = 973), laboratory measurement data (*n* = 93), anthropometric measurements (*n* = 174), and demographic data (*n* = 62). Finally, we retained 1216 participants who were aged ≥60 years and for whom complete data were available (Fig. 1).

### Data collection

We collected the demographic characteristics of the participants, such as age, sex, educational qualifications, history of smoking or alcohol consumption, physical exercise status, and financial status. This was done using a questionnaire.

The age was taken as a continuous variable and sex as a binary variable (male or female). We converted the years of education into a binary variable (< 1 year of schooling or ≥ 1 year of schooling). The smoking status, alcohol drinking status, exercise status, and financial status were binary variables (To be answered yes or no). We collected information by using questions such as “Do you smoke at the present time,” “Do you drink alcohol at the present time,” “Do you do exercises regularly at present,” and “Does all of your financial support sufficiently pay your daily costs”.

### Anthropometric assessment

In our survey, we measured the height (in meters) and weight (in kilograms) of the participants; we calculated body mass index (BMI) by dividing the weight (in kgs) by the square of height (m^2^). The participants were advised to relax their body and a measuring tape was used to measure the CC and waist circumference (WC) of each participant.

### Laboratory investigations

Professional nurses collected blood samples from participant who had fasted overnight. Samples underwent routine hematological examination (including white blood cells, lymphocyte, and hemoglobin [Hb]) in the local center for disease control and prevention. Serum samples were analyzed for lipid profile (total cholesterol, high-density lipoprotein cholesterol [HDL-C], and triglycerides [TG]), plasma albumin (Alb), vitamin D3 (Vit D3), vitamin B12 (Vit B12), and high-sensitive C-reactive protein (HS-CRP) at the central clinical laboratory at Capital Medical University in Beijing. The neutrophil-lymphocyte ratio (NLR) was calculated as (white blood cells – lymphocyte)/lymphocyte as given in the literature [13].

### Frailty diagnosis criteria

Frailty status was measured by the FI that was similar to previous CLHLS studies [14, 15]. The FI included routine activities of daily living, instrumental activities of daily living, cognitive functions, overall health status, emotion, and the presence of specific diseases. We had 39 items and 40 health deficits (details in Supplementary Table 1). FI was defined as 0 or 1 for terms. We scored a deficit as 2 if the participants had a serious illness or had been bedridden during the past 2 years. We added the observed number of deficits and then divided the sum by the total number of deficits included. We also defined the FI in two groups based on the literature [16] as non-frail (FI < 0.25) and frail (FI ≥ 0.25).

### Statistical analyses

The demographic characteristics (age, sex, education, history of smoking and alcohol consumption, exercise status, and financial status), anthropometric assessment (BMI, CC, and WC) and laboratory results (Alb, Hb, Vit D3, Vit B12, cholesterol, HDL-C, TG, NLR, and HS-CRP) were analyzed for two groups (non-frailty and frailty). We used Student’s t test for continuous variables and Wilcoxon Mann–Whitney test for categorical variables. To explore the risk factors for frailty, multiple logistic regression analysis was performed. We adjusted for demographic characteristic variables (model 1), then adjusted for laboratory measurement variables (model 2), and finally the model was further adjusted for all variables (model 3). In order to predict frailty, receiver operating characteristic (ROC) curve was used to find cut-off points of CC. We established a prediction model for frailty by binary logistic regression. We used the SPSS 26.0 (SPSS Inc., Chicago, Illinois, USA) software for statistical analysis. We considered it to be statistically significant if *P* values were less than 0.05. We provided odds ratio (OR) with 95% confidence interval (CI) as well.

## Results

### Characteristics of participants

**Fig. 2:**
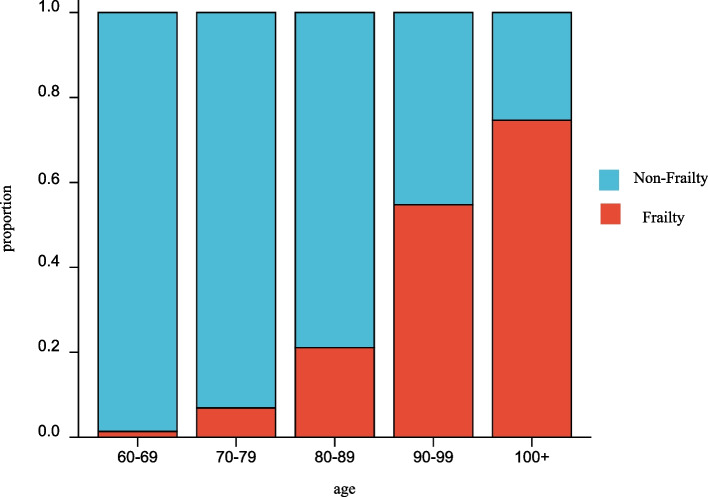
The proportion of frailty by age group in the study sample

**Fig. 3:**
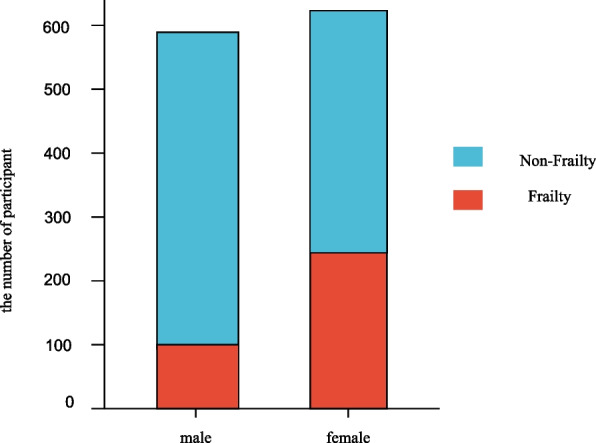
The prevalence of frailty by sex in the study sample

In our study, we enrolled 1216 participants and divided them into two groups. 874 (71.9%) were categorized as non-frail and 342 (28.1%) as frail at baseline. The characteristics of subjects are presented in Table 1. The age of the participants was 83.45 ± 11.41 years. We further divided the participants into five groups according to their age and found that as people got older, the incidence of frailty increased in the population (Fig. 2.) We found that the proportion of frailty was higher in women than in men (Fig. 3.) We discovered that age (*p*<0.001), sex (*p <* 0.001), smoking habit (*p <* 0.001), alcohol consumption (*p <* 0.001), exercise (*p <* 0.001), education (*p <* 0.001), BMI (*p <* 0.001), Alb (*p* < 0.001), Hb (*p* < 0.001), Vit D3 (*p* < 0.001), TG (*p* = 0.014), and HS-CRP (*p* = 0.002) factors were significantly different between the two groups. However, financial status and values of WC, Vit B12, Cholesterol, HDL-C, and NLR were not significantly different between the two groups.

**Table 1:** The characteristics of non-frail and frail

Characteristics	non-Frailty (*n* = 874)	frailty (*n* = 342)	statistics	*p*
Age, mean (SD), years	79.65 ± 10.05	93.15 ± 8.59	*t* = − 23.46	<0.001
Sex, n (%)			χ2 = 78.28	<0.001
male	497 (56.9%)	98 (28.7%)		
female	377 (43.1%)	244 (71.3%)		
Smoke, n (%)	185 (21.2%)	27 (7.9%)	χ2 = 30.08	<0.001
Alcohol n (%)	171 (19.6%)	33 (9.6%)	χ2 = 17.31	<0.001
Exercise, n (%)	176 (20.1%)	29 (8.5%)	χ2 = 23.83	<0.001
Education, n (%)	448 (51.3%)	66 (19.3%)	χ2 = 102.90	<0.001
Financial status,n (%)	756 (86.5%)	286 (83.6%)	χ2 = 1.66	0.203
BMI,mean (SD), cm	22.30 ± 3.26	20.90 ± 4.01	*t* = 5.66	<0.001
WC, mean (SD), cm	82.22 ± 9.50	81.50 ± 10.10	*t* = 1.13	0.257
Alb, mean (SD), g/L	43.48 ± 3.27	41.11 ± 4.02	*t* = 9,73	<0.001
Hb, mean (SD), g/L	130.42 ± 17.43	120.83 ± 18.41	*t* = 8.49	<0.001
Vit D3	44.26 ± 19.46	34.61 ± 17.90	*t* = 8.24	<0.001
Vit B12	425.10 ± 196.75	402.80 ± 195.80	*t* = 1.78	0.075
CHOL, mean (SD), mmol/L	4.80 ± 1.01	4.80 ± 0.97	*t* = −0.101	0.920
HDL-C, mean (SD), mmol/L	1.42 ± 0.37	1.40 ± 0.37	*t* = 0.981	0.327
TG, mean (SD), mmol/L	1.31 ± 0.83	1.20 ± 0.63	*t* = 2.47	0.014
NLR, mean (SD)	2.37 ± 1.19	2.49 ± 1.34	*t* = −1.51	0.131
HS-CRP, mean (SD), mg/l	2.36 ± 5.01	3.62 ± 6.77	*t* = −3.11	0.002

### Relationship between calf circumference (CC) and frailty

**Fig. 4:**
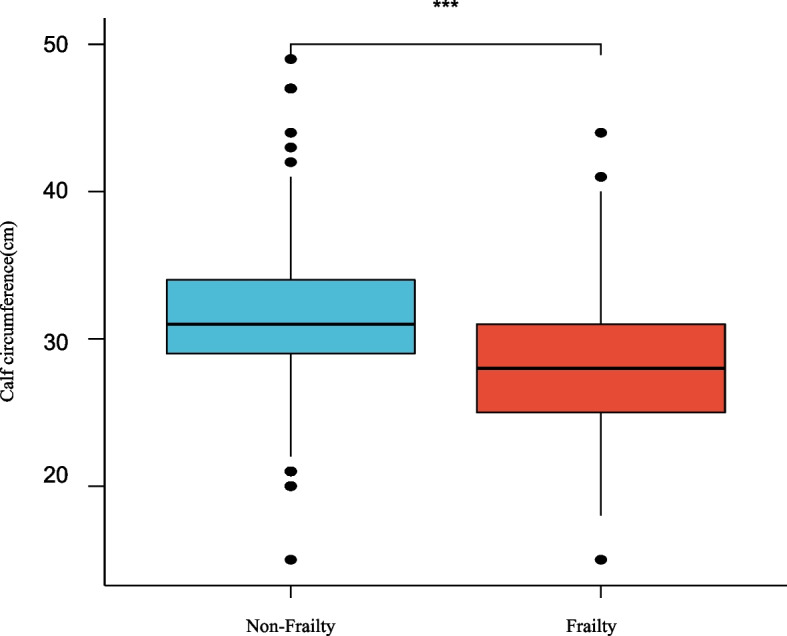
Relationship between CC and frailty in two groups

The mean calf circumference was 31.54 ± 4.16 cm in non-frail participants and 28.04 ± 4.53 cm in frail participants. The values were significantly different between the two groups (*p* < 0.001) (Fig. 4).

### Factors associated with frailty

To explore which factors may affect the frailty status, we used multiple logistic regression analysis and built 3 models (Table 2). The model 1 adjusted for age, sex, smoking habits, alcohol consumption, exercise, education, and BMI. The model 2 adjusted for Alb, Hb, Vit D3, TG, and HS-CRP. Finally, model 3 adjusted for all potential factors. The results showed that age (OR = 1.117,95% CI: 1.095–1.139, *P <* 0.001), exercise (OR = 0.601, 95% CI: 0.366–0.984, *P* = 0.043), CC (OR = 0.947, 95% CI: 0.904–0.993, *P* = 0.023), Alb (OR = 0.926, 95% CI: 0.883–0.972, *P* = 0.002), and Vit D3 levels (OR = 0.985, 95% CI: 0.976–0.995, *P* = 0.002) were independent risk factors.

**Table 2:** The factors for frailty by multiple logistic regression analysis

Characteristics	Model 1	*P*	Model 2	*P*	Model 3	*P*
	OR(95%CI)		OR(95%CI)		OR(95%CI)	
Age	1.127 (1.106,1.149)	<0.001			1.117 (1.095,1.139)	<0.001
Sex	0.766 (0.518,1.132)	0.181			0.838 (0.550,1.277)	0.411
Smoke	0.693 (0.414,1.159)	0.162			0.656 (0.388,1.109)	0.116
Alcohol	0.688 (0.410,1.089)	0.106			0.662 (0.403,1.088)	0.104
Exercise	0.596 (0.366,0.969)	0.037			0.601 (0.366,0.984)	0.043
Education	0.865 (0.582,1.286)	0.474			0.833 (0.557,1.245)	0.372
BMI	1.018 (0.971,1.067)	0.467			1.020 (0.972,1.071)	0.417
CC	0.943 (0.900,0.988)	0.013	0.863 (0.833,0.895)	<0.001	0.947 (0.904,0.993)	0.023
Alb			0.880 (0.843,0.918)	<0.001	0.926 (0.883,0.972)	0.002
Hb			0.991 (0.983,0.999)	0.033	1.004 (0.994,1.014)	0.403
Vit D3			0.980 (0.972,0.989)	<0.001	0.985 (0.976,0.995)	0.002
TG			0.944 (0.778,1.145)	0.559	1.011 (0.811,1.259)	0.924
HS-CRP			1.006 (0.983,1.030)	0.612	1.003 (0.978,1.029)	0.808

Model 1 was adjusted for age, sex, smoking habits, alcohol consumption, exercise, education, and BMI

Model 2 was adjusted for Alb, Hb, Vit D3, TG, and HS-CRP

Model 3 was adjusted for all potential factors

### The cut-off value of CC for frailty

**Fig. 5:**
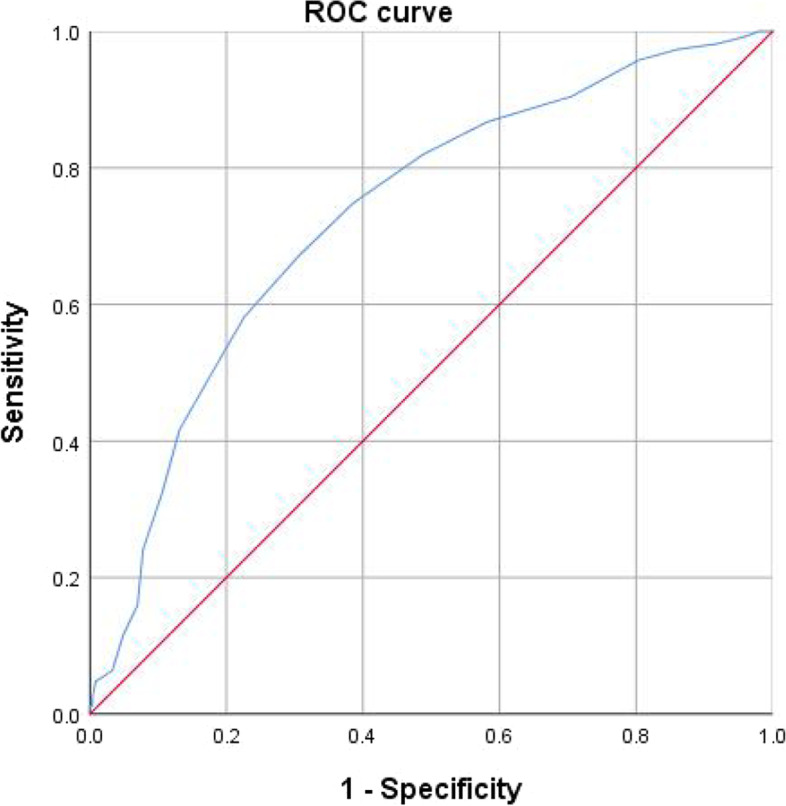
ROC curve analysis of CC for frailty in women. AUC = 0.732 (95% CI: 0.691–0.772; *P* < 0.001); CC cut-off point in women = 28.5 cm; Youden index = 0.365; Sensitivity: 66.8%; Specificity: 69.7%

**Fig. 6:**
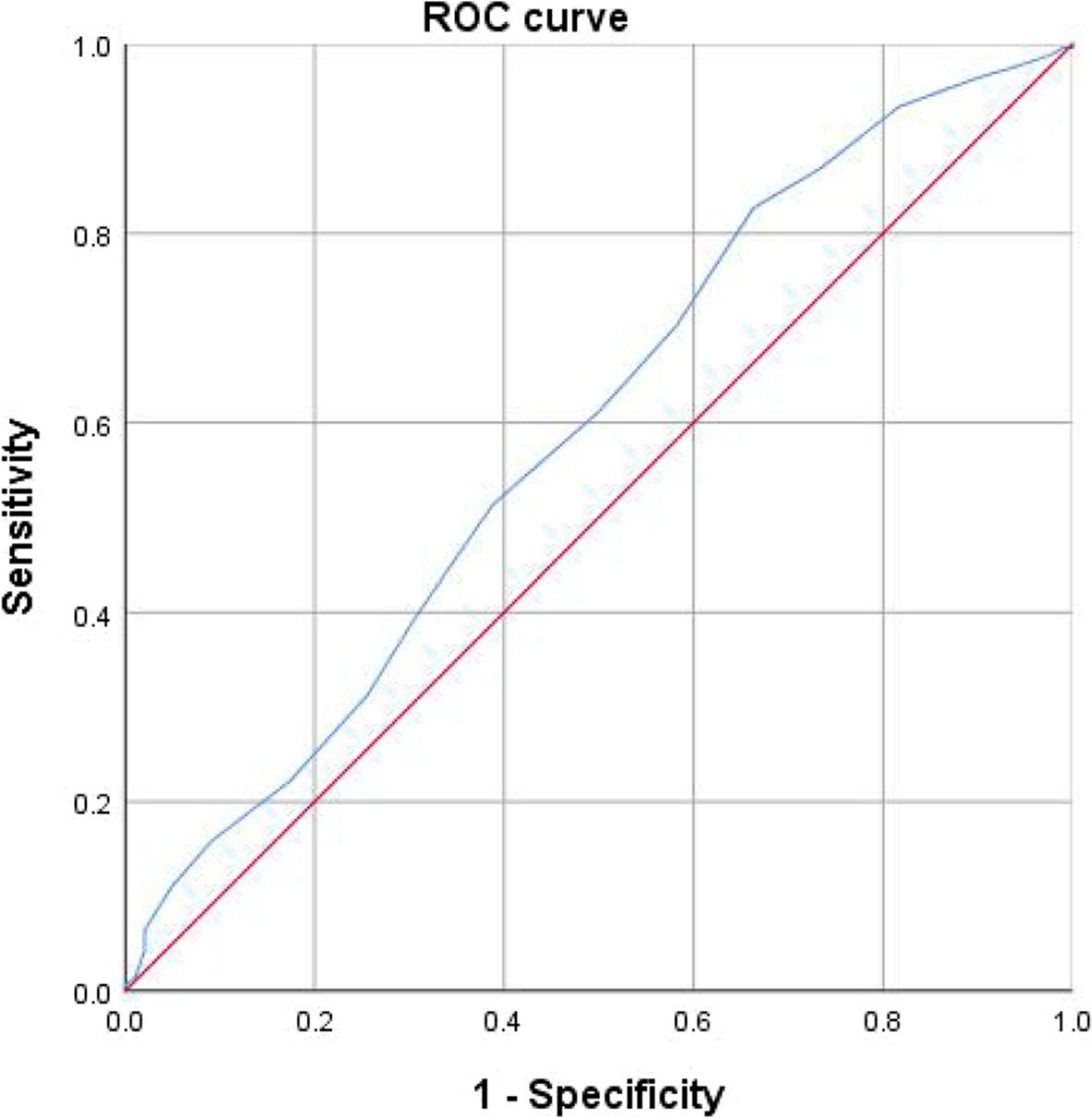
ROC curve analysis of CC for frailty in men. AUC = 0.592 (95% CI: 0.528–0.656; *P* = 0.004); CC cut-off point in men = 29.5 cm; Youden index = 0.164; Sensitivity: 82.7%; Specificity: 33.7%

We used ROC curve analysis to determine the best cut-off value of CC to predict frailty. In women,the best cut-off value of CC was 28.5 cm. The AUC was 0.732 (95% CI: 0.691–0.772; *P* < 0.001); and the Youden index was 0.365; the sensitivity was 66.8% and the specificity was 69.7% (Fig. 5). The CC of 29.5 cm was considered the best cut-off value in men. The area under the curve (AUC) was 0.592 (95% CI: 0.528–0.656, *P* = 0.004) and the Youden index was 0.164; the sensitivity was 82.7% and the specificity was 33.7%. (Fig. 6).

We created a simple prediction model for frailty that contained age,sex and CC by Binary Logistic Regression:

$$\textrm{Logit}(P)=-9.756+0.123\ast \textrm{Age}-0.512\ast \textrm{Sex}\left(\textrm{man}=1,\textrm{woman}=0\right)-0.053\ast \textrm{CC},P={e}^{\textrm{logit}}(P)/1+{e}^{\textrm{logit}}(P).$$

ROC curve analysis found that the AUC was 0.849.

(95% CI:0.826–0.872, *P* < 0.001) (Supplementary Fig. 1).

## Discussion

In our study, we observed that CC and frailty are closely correlated in Chinese adults aged over 60 years. In addition, CC is an easy and helpful indicator to predict frailty in community-dwelling old adults. The best cut-off value was 29.5 cm in man and 28.5 cm in women.

China is one of the most rapidly aging societies in the world and frailty is a common geriatric syndrome in old adults. We found that the prevalence of frailty were higher in women than in men, which was consistent with previous literature [17]. We used FI to assess frailty, as consistent with previous literature [14]. FI is a deficit accumulation model and counts at least 30 health deficits. In our research, FI contained indices involving daily living, function, cognition, health, emotion, and diseases, which are more comprehensive compared to the fried phenotype. However, the cutoffs of FI are controversial; we defined FI ≥0.25 as frail in accordance with previous literature [16].

Researchers have recently started to pay close attention to CC. It is not only a useful predictor of sarcopenia in patients with liver disease [18], but also an independent predictor of mortality in cancer patients [19]. Moreover, CC can predict nutritional risks in hospitalized patients [11].

Our study, is the first to suggest that CC may predict frailty in elder community. Frailty is associated with various factors and the causative mechanism is complex and still unclear [20]. Up till now, we have no precise and easy method to measure frailty. In this study, we discovered that people categorized as frail had low CC. We then adjusted for confounding factors and built three models. CC was a significant indicator of frailty in older people. In the future, we used to build a model using age、sex and CC measurements to predict frailty.

The Asian Working Group for Sarcopenia [5] diagnosed sarcopenia using CC measurements (men < 34 cm or women < 33 cm). Using ROC analsis, we found that the best cut-off value of CC to predict frailty was < 29.5 cm for men and < 28.5 cm for women respectively. In addition, as mentioned in earlier studies, BMI ≥30 kg/m^2^ and waist circumference > 102 cm for men or > 88 cm for women were associated with increased frailty among old people [21]. However, there was no association between WC or BMI and frailty in our study. The results are different from previous studies, perhaps because of the mean age of the participants studied is different or else because FI may have been defined differently in the earlier studies.

The mechanics of frailty are complex, and seem to be related to inflammatory cytokines, nutrient status, vitamin levels, and other factors [4, 22]. Based on our findings, the blood indices of Alb and Vit D3 had statistical significance, similar to the results obtained in earlier studies [23]. However, in our data, inflammatory cytokines, including NLR and HS-CRP had no statistical significance. In the future, we may focus on more biological markers to explore potential underlying mechanisms of the frailty syndrome.

Our study was a large cohort study among older communal adults, utilizing face-to-face interviews. However, several limitations associated with the study must be mentioned. First, the study design did not define the standard method for measuring the CC. Second, there was no consensus regarding which leg was to be measured; the result may have been influenced by whether or not the dominant leg was measured. Moreover, all participants were from China. Therefore, we may not be able to extrapolate our findings to the elderly population of other countries. Studies involving more participants from across the world may be needed.

## Conclusions

In summary, our research confirms the association between CC and frailty syndrome in old adults living in Chinese communities. The CC is an easily measurable and convenient marker of frailty.

## Supplementary Information

**Additional file 1.**

## Data Availability

The datasets are publicly available from Chinese Longitudinal Healthy Longevity Survey (CLHLS) and can be downloaded in https://opendata.pku.edu.cn/dataverse/CHADS. The research used the seventh wave of the CLHLS conducted in 2014.

## Abbreviations

CC: calf circumference
FI: frailty index
WC: waist circumference
Alb: plasma albumin
Vit D3: vitamin D3
Vit B12: vitamin B12
CLHLS: Chinese Longitudinal Healthy Longevity Survey
BMI: body mass index
ROC: receiver operating characteristic
HDL-C: high-density lipoprotein cholesterol
NLR: neutrophil-lymphocyte ratio
TG: triglyceride
CI: confidence interval
OR: odds ratio
HS-CRP: high-sensitive C-reactive protein
Hb: hemoglobin
