# Supplementary material for: Calf circumference predicts frailty in older adults: the Chinese longitudinal healthy longevity survey
Source: BMC Geriatr. 2022 Dec 5;22:936. doi: 10.1186/s12877-022-03644-w (PMC9720947; doi:10.1186/s12877-022-03644-w)
Supplement: Supplementary file 1 — Additional file 1. [file 12877_2022_3644_MOESM1_ESM.docx]

Supplementary Table 1:The list of items included in calculating the FI

| Classification | Item |
| --- | --- |
| Instrumental activities of daily living | 1.IADL: Unable to visit neighbors by oneself |
|  | 2 IADL: Unable to shop by oneself if necessary |
|  | 3 IADL: Unable to cook meals by oneself if necessary |
|  | 4 IADL: Unable to wash clothing by oneself |
|  | 5 IADL: Unable to walk continuously for 1 km |
|  | 6 IADL: Unable to lift a weight of 5 kg (such as a heavy bag of groceries) |
|  | 7 IADL: Unable to continuously crouch and stand up 3 times |
|  | 8 IADL: Unable to use public transportation |
| Function | 9 Functional limitations: Unable to put hand behind neck |
|  | 10 Functional limitations: Unable to put hand behind lower back |
|  | 11 Functional limitations: Unable to raise arm upright |
|  | 12 Functional limitations: Unable to stand up from sitting in a chair |
|  | 13 Functional limitations: Unable to pick up a book from the floor |
| Activities of daily living | 14 ADL: Needs assistance bathing |
|  | 15 ADL: Needs assistance dressing |
|  | 16 ADL: Needs assistance toileting |
|  | 17 ADL: Needs assistance in indoor transferring |
|  | 18 ADL: Needs assistance eating |
|  | 19 ADL: Incontinence |
| Cognition | 20 Cognitively impaired (based on the MMSE) |
| Health status | 21 Poor self-rated health |
|  | 22 Health worsened in the past year |
|  | 23 Poor interviewer-rated health |
|  | 24 Hearing loss |
|  | 25 Vision loss |
|  | 26 Abnormal heart rhythm |
| Emotion | 27 Symptom of psychological distress (based on loneliness, usefulness,  fearfulness) |
| Diseases | 28 Number of serious illnesses in the past 2 years |
|  | 29 Suffering from hypertension |
|  | 30 Suffering from diabetes |
|  | 31 Suffering from tuberculosis |
|  | 32 Suffering from heart disease |
|  | 33 Suffering from stroke/cerebrovascular disease |
|  | 34 Suffering from bronchitis, emphysema, asthma, or pneumonia |
|  | 35 Suffering from cancer |
|  | 36 Suffering from arthritis |
|  | 37 Suffering from bedsores |
|  | 38 Suffering from gastric or duodenal ulcers |
|  | 39 Suffering from Parkinson’s disease |

item28: Persons reporting two or more illnesses are assigned a value of 2.

Supplementary Figure 1: ROC curve analysis of prediction model for frailty.


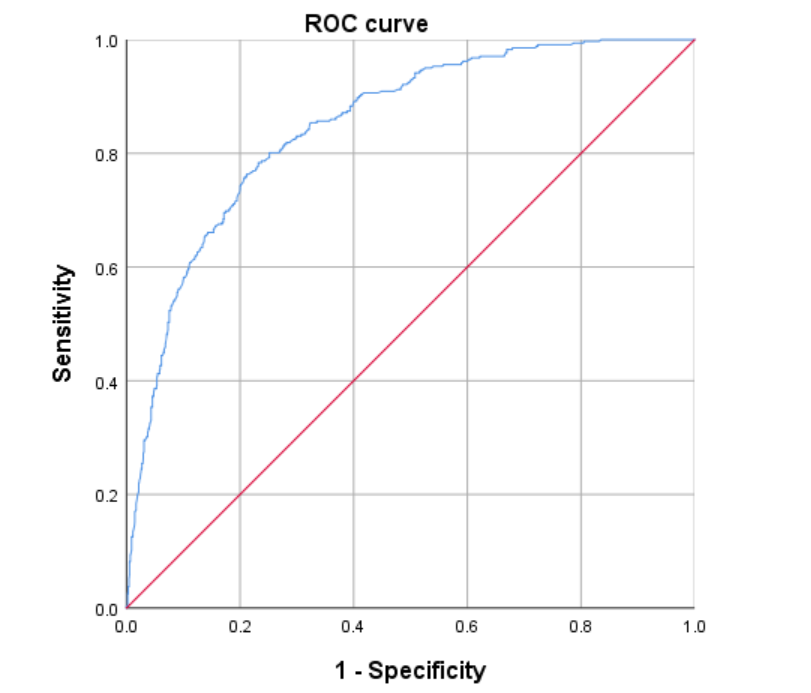


AUC = 0.849 (95% CI：0.826–0.872, *P* < 0.001)
